# The VITAAL Stepping Exergame Prototype for Older Adults With Major Neurocognitive Disorder: A Usability Study

**DOI:** 10.3389/fnagi.2021.701319

**Published:** 2021-11-04

**Authors:** Nathalie Swinnen, Eling D. de Bruin, Chantal Dumoulin, Melanie Thalmann, Vânia Guimarães, Jacqueline De Jong, Mathieu Vandenbulcke, Davy Vancampfort

**Affiliations:** ^1^Department of Rehabilitation Sciences, KU Leuven, Leuven, Belgium; ^2^Department of Health Sciences and Technology, Institute of Human Movement Sciences and Sport, ETH Zürich, Zurich, Switzerland; ^3^Division of Physiotherapy, Department of Neurobiology, Care Sciences and Society, Karolinska Institute, Stockholm, Sweden; ^4^Faculty of Medicine, University of Montreal, Montreal, QC, Canada; ^5^Montreal University Geriatrics Institute, Montreal, QC, Canada; ^6^Fraunhofer Portugal Research Center for Assistive Information and Communication Solutions, Porto, Portugal; ^7^Physio SPArtos, Interlaken, Switzerland; ^8^University Psychiatric Centre, KU Leuven, Leuven, Belgium; ^9^Department of Neurosciences, KU Leuven, Leuven, Belgium

**Keywords:** active videogame, dementia, feasibility, motivation, physical activity, residential care, serious game

## Abstract

**Purpose:** This study investigates the usability of a stepping exergame in older adults with major neurocognitive disorder (MNCD) residing in a long-term care facility.

**Materials and Methods:** A mixed methods study was conducted. Participants played exergames for 30 min on one try-out session. During the exergames, the think aloud method was used, and field notes were taken by the facilitator. Following the exergames, participants completed the System Usability Scale (SUS) and a semi-structured in-depth interview about usability including their personal experiences. Audio files were transcribed and a thematic content analysis of the think aloud data, field notes and interviews were performed using NVivo 12.

**Results:** Twenty-two participants with MNCD were included [mean age = 84.3 ± 5.5 (70–95) years; 81.8% women; Short Physical Performance Battery score = 7.5 ± 3.2 (1–12), Montreal Cognitive Assessment score = 11.9 ± 4.4 (2–19)]. System usability was rated “ok to good” with a mean SUS score of 57.8 (*SD* = 12.3) with scores ranging from 37.5 to 90.0. Five main themes emerged from the thematic content analysis: (1) perceived user friendliness and acceptability of the exergames; (2) interactional experience; (3) motivational factors; (4) training modalities; and (5) risks. There were no adverse events nor dropouts.

**Conclusion:** Participants evaluated the usability of the exergames positively. The results indicate that the stepping exergame is usable in older adults with MNCD.

## Introduction

The number of older adults with major neurocognitive disorder (MNCD) is increasing, primarily driven by population aging (Alzheimer’s Disease International, 2019). MNCD is a clinical syndrome marked by cognitive decline, motor deficits and psychological and behavioral problems (LoGiudice and Watson, 2014). Older adults with MNCD often require added assistance with their activities of daily living (Arvanitakis et al., 2019) and this can ultimately lead to the displacement to a long-term care facility (Forbes et al., 2015). This is imposing a compelling burden on health care systems and has resulted in MNCD being considered a global public health priority (WHO, 2016). The burden of MNCD on health care systems is further compounded by a high risk of falling and associated injuries and disability (Sharma et al., 2018).

In order to reduce the risk of falling in older adults with MNCD residing in long-term care facilities, physical activity should be an important component of the multidisciplinary approach (Forbes et al., 2015; Vancampfort et al., 2020). There is compelling evidence that physical activity improves strength, endurance, balance, gait stability, gait speed, and overall wellbeing in older adults with MNCD (Forbes et al., 2015; Groot et al., 2016; Lam et al., 2018). Currently, clinical practice guidelines do not refer to combined cognitive and physical training programs (Laver et al., 2016; Shaji et al., 2018). This is surprising as not only a decline in physical functions is responsible for gait impairments and higher risks of falls, but also impaired cognitive performance including impairments in executive functioning (Holtzer et al., 2006; Yogev-Seligmann et al., 2008; de Bruin and Schmidt, 2010; Segev-Jacubovski et al., 2011; Mirelman et al., 2012). More recently, the prevalence of coexisting physical limitations and cognitive decline, described as motoric cognitive risk syndrome, has been estimated to be 10% in aging adults (Meiner et al., 2020). To slow down the cognitive and physical decline, and to prevent falls, combined motor-cognitive interventions which are adapted to the participants’ individual needs might be useful (Pichierri et al., 2011; Bamidis et al., 2014; Eggenberger et al., 2015). A promising option for such a simultaneous cognitive-motor training is exergame training (de Bruin et al., 2010). Exergames are videogames that require movement in order to play the games (Stanmore et al., 2017). Previous studies have found that exergaming improves gait speed, mobility, balance, and cognitive functions, and reduces apathy and fear of falling in older adults with MNCD (Dietlein et al., 2018; Swinnen et al., 2020; Robert et al., 2021). Another advantage is that exergames are engaging and might overcome low adherence rates that are often reported in physical interventions for this population (Forbes et al., 2015; Ben-Sadoun et al., 2016).

Stepping exergame training is feasible and engaging in older adults with MNCD in long-term care facilities (Swinnen et al., 2020). Stepping exergames require participants to stand upright and perform steps, which directly addresses gait and balance (Kappen et al., 2018). Exergaming in an upright standing body position also enhances processing speed and attentional selectivity (Rosenbaum et al., 2017) and influences visual working memory performance (Dodwell et al., 2019). However, compared to seated cognitive games, exergames might impose a higher risk of falling than seated exergames. Currently, safe stepping exergame programs designed for older adults that are portable and affordable are still lacking. In order to fill this gap, an international research group developed a prototype of an individualized multicomponent stepping exergame training solution for geriatric rehabilitation (VITAAL, 2020). This project, entitled VITAAL, was launched in May 2018 and is funded by the European Commission as a part of the Active Assisted Living Program (AAL Association, 2020). The developed solution consists of two wearable sensors and a web-based interface that allows a direct follow-up and data processing by healthcare professionals. The system aims to provide evidence-based motor-cognitive training with high usability and easy setup in the clinic and at home.

However, in order to develop a user-friendly and acceptable training solution, end user involvement is required. Older adults with MNCD are often still able to communicate their opinions about what is important to them (Cahill and Diaz-Ponce, 2011). Researchers have previously recommended an end user participatory design with direct involvement of older adults with MNCD throughout the whole development process (Meiland et al., 2012). It has been highlighted that older adults with MNCD can contribute to establishing technological solutions that support them in the self-management of their symptoms and challenges in daily living, as well as contribute to the development by providing useful feedback, also in long-term care facilities (Span et al., 2013; Kort et al., 2019).

Therefore, the aim of this study is to investigate the usability of the VITAAL exergame prototype through a mixed methods design that combines observations, the think aloud approach, semi-structured interviews, and a system usability scale in institutionalized older adults with MNCD. The combination of both quantitative and qualitative data provides a full picture of the users’ perspectives. A secondary aim is to investigate whether, and to what extent, the variance in the system usability score can be predicted by the variance in age, gender, cognitive functioning, and lower extremity functioning in institutionalized older adults with MNCD.

## Materials and Methods

A mixed methods design was used. The Consolidated criteria for reporting qualitative research (COREQ) framework was implemented (Tong et al., 2007). The trial was registered in ClinicalTrials.gov (Identifier: NCT04664920).

### Participants and Procedure

Over a period of 1 month, all residents of long-term care facility de Wingerd in Leuven, Belgium, with MNCD were screened for inclusion. Possible causes of major neurocognitive disorder eligible for inclusion were vascular dementia, Alzheimer’s disease, mixed dementia, Parkinson’s disease, or Lewy body disease, as well as unspecified, as stated by the criteria of the fifth edition of the Diagnostic and Statistical Manual of Mental Disorders (DSM 5) (American Psychiatric Association, 2013). Diagnoses were made by the treating psychiatrist. Additional inclusion criteria were age ≥60 years; visual acuity with correction sufficient to work with a TV screen; a minimum stay of 2 weeks in the long-term care facility at the time of inclusion and being physically capable of doing stepping exercises. Subjects manifesting one or more of the following criteria were excluded from the study: any unstable health condition which, according to the American College of Sports Medicine Standards, might lead to unsafe participation (ACSM); and mobility impairments that didn’t allow to play the exergame. All eligible participants played the exergame for 30 min on one try-out session. During the exergame performance, the think aloud method (Ratcliffe et al., 2019) was used, and field notes were taken by the observer. After the exergame performance, participants completed the System Usability Scale (Brooke, 1996) and a semi-structured in-depth interview concerning the usability of the device. To describe the population more in detail, participants completed the Montreal Cognitive Assessment (MoCA) (De Roeck et al., 2019) and the Short Physical Performance Battery (SPPB) (Guralnik et al., 2000; Fox et al., 2014) prior to the exergame. Their comorbidities, indoor mobility, fear of falling, and level of physical activity prior to participation were investigated. The protocol was approved by the Medical Ethics committee of UZ Leuven (registration: S63304/B322201941828). Written informed consent was obtained from the participants according to the Declaration of Helsinki. No compensation for participation was given.

### VITAAL Exergame Prototype Session

**FIGURE 1:**
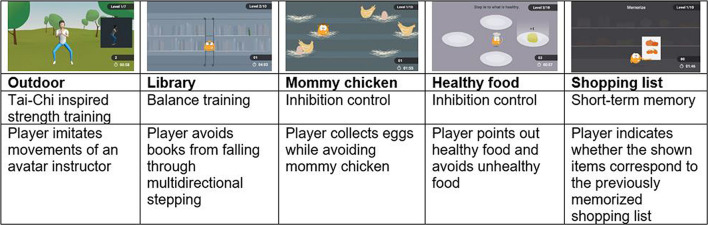
Game description.

**FIGURE 2:**
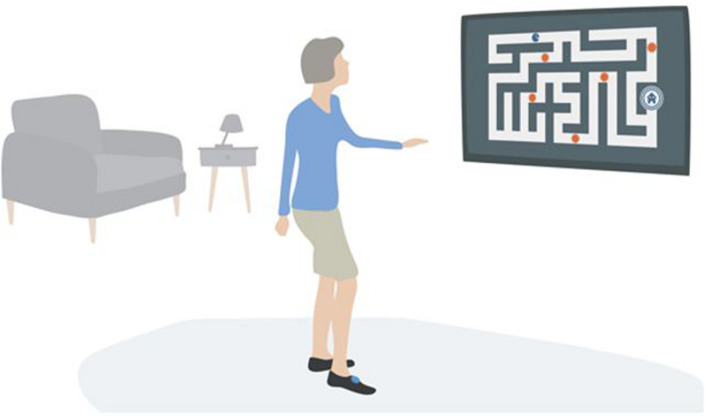
System set-up.

Participants individually performed one single exergame session using the VITAAL prototype, which is an innovative, comprehensive system for geriatric rehabilitation and treatment in geriatric healthcare. The VITAAL solution implements personalized training programs based on interventions for mobility impairment, urinary incontinence, and cognitive impairment. The exergame mainly consists of three components: strength training, balance training, and cognitive training (VITAAL, 2020). For strength training, a combination of classical strength exercises and Tai Chi-inspired movements are included. Since Tai Chi is mainly performed in a semi-squat posture, a large load is placed on the muscles of the lower extremities. For balance training, step-based training is included, as the execution of rapid and well directed steps has been shown to be effective in preventing falls (Kattenstroth et al., 2013; Merom et al., 2013; Okubo et al., 2016). Both Tai Chi-inspired exercises and step-based exercises, combined with challenging game tasks, provide a holistic training requiring motor functions, cognition, and mental involvement (Gajewski and Falkenstein, 2016; Lim et al., 2019). Some cognitive training is already included in these training components as they represent simultaneous cognitive-motor interaction and require motor and cognitive functions. Specific attentional and executive functions are important for walking abilities and safe gait (Holtzer et al., 2006; Yogev-Seligmann et al., 2008; de Bruin and Schmidt, 2010; Segev-Jacubovski et al., 2011; Mirelman et al., 2012). Therefore, the VITAAL exergame explicitly targets these neuropsychological functions (selective attention, divided attention, inhibition/interference control, mental flexibility, working memory). See Figure 1 for an overview of the different minigames and the focus of training per game. To maximize the benefits for participants, the exergame implements some basic general training principles: feedback, optimal load of task demands, progression of difficulty and high variability (Healy et al., 2014). The system set-up was developed to be easily applied with limited technical equipment and knowledge in long-term care facilities or in clinics. As a web-based exergame, it is designed to run anywhere if there is a Bluetooth and internet-enabled device connected with a screen (e.g., PC, laptop, tablet, etc.). The front-end is designed for large screens and is ideally visualized on a TV screen. The system is supported by a backend (main server supporting the whole service and data storage), a web portal (with information about interventions, sessions, results per session or over a specific period, etc.) and two wearable inertial sensors for measuring the stepping movements and game navigation. The web portal enables a follow-up of the personalized training intervention and provides relevant data in the rehabilitation process for researchers or healthcare providers. The two inertial sensors are placed on the shoes and are capable of sensing accelerations and angular rotations caused by movement. They communicate via Bluetooth with the software running on the web-enabled device. Participants played all the minigames that were available in this prototype, namely a minigame focusing on Tai Chi-inspired strength training (i.e., ‘outdoor’), two minigames focusing on balance training (i.e., ‘library’ and ‘mommy chicken’), a minigame focusing on inhibition control (i.e., ‘healthy food’), and an minigame focusing on short-term memory (i.e., ‘shopping list’). The design and development of these VITAAL minigames considered inputs from older adults, resulting from the investigation phase of the project (AAL Association, 2020), from the feedback obtained in a previous study (Guimarães et al., 2018), and from a multidisciplinary team, including movement scientists, clinicians and game designers. Movement scientists and clinicians agreed that an exergame mostly based on the execution of multidirectional steps would fit the needs of the target population the best. Users should also be able to perform multidirectional steps while responding to specific cognitive tasks, or contracting the pelvic floor muscles, which could largely improve the outcomes of the training. Considering that most daily life activities require simultaneous performance of physical and cognitive functions, combining physical and cognitive exercises in a single exergame solution could potentially boost the benefits of the exergames (Sauro, 2011). The design team on its turn aimed to motivate and engage the player by balancing challenge and fun as follows: (i) distributing types and number of exercises by several minigames with different scenes and goals to promote variety and avoid monotony, (ii) adapting the difficulty level of each game according to the individual in-game progression in order to prevent frustration and foster learnability (although this option is not yet available in the current prototype), and (iii) providing one single instruction and focus at a time to avoid an overwhelming experience (Sauro, 2011). The participants played the exergames autonomously and the facilitator (a physiotherapist) only intervened when help was required. An example of the system set-up is included in Figure 2.

### System Usability Scale

After the exergame session, the System Usability Scale (SUS) was completed (Brooke, 1996). It is a commonly used scale for exergame evaluation and provides a global view of subjective usability of a product or a system. The SUS consists of ten questions/items which are scored on a 5-point Likert scale, ranging from 1 (strongly disagree) to 5 (strongly agree). In the SUS, five questions have a rather negative connotation and five have a positive connotation. The evaluation results in a total score, provided in a scale from 0 to 100. The score is calculated by subtracting one from the user responses for items with a positive connotation (items 1, 3, 5, 7, and 9) and by subtracting five from the user responses for items with a negative connotation (2, 4, 6, 8, and 10). This scales all values from 0 to 4, with 0 being the most negative and 4 the most positive response. The converted responses were multiplied by 2.5 to convert the scale from 0 to 100. SUS scores below 25 correspond to a worst imaginable system; scores from 25 to 39 correspond to worst imaginable to poor; scores from 39 to 52 correspond to ok; from 52 to 73 correspond to ok to good; scores from 73 to 85 correspond to good to excellent and from 85 to 100 corresponds to excellent to best imaginable (Sauro, 2011). The SUS is reliable and valid in non-clinical adults (Brooke, 1996; Tullis et al., 2008). Previous studies have reported internal reliability with Cronbach’s alpha values between 0.79 and 0.97 (Finstad, 2010; Dianat et al., 2014). The convergent validity with other measures of perceived usability were acceptable. Regarding exergames, SUS provides information on whether older adults are confident playing the minigames, whether they desire to use the exergames frequently, and whether the exergames are easy to use for cognitive training and physical activity.

### Think Aloud Method

Participants were encouraged to explain their views and experiences during exergaming through a think aloud approach (Ratcliffe et al., 2019). The think aloud approach is a common observational technique for eliciting insight into the users’ thinking process while actively performing a task (Eccles and Arsal, 2017; Ratcliffe et al., 2019). Participants were encouraged to say everything that came to their mind while performing the exergame activities. Field notes were taken during and after exergaming performance to complement the information gained from the think aloud approach. The observer wrote down the user actions for each of the tasks, as well as all the problems that occurred. It has been previously demonstrated that the think aloud method is an appropriate method to engage older adults with MNCD as co-creators of solutions that accommodate to their needs (Kort et al., 2019).

### Semi-Structured Interview Regarding the Participants’ Exergame Experiences

A semi-structured interview was executed after the exergame to acquire the participants’ experiences with the exergame. The interview focused on the qualitative evaluation of the user’s gameplay experiences related to the body movements and the virtual game scenario. During the interview, no notes were taken in order to fully focus on the participants’ verbal and non-verbal communication. The recorded interviews lasted between 3 and 11 min (mean 6 min). The interview guide is included in Supplementary Material 1. The interviewer was also the person that observed the exergame session. Therefore, the interviewer and participant were familiar with each other. Every interview was recorded and fully transcribed to a written form. Transcripts were not returned to participants for comment or correction. Guidelines for ethical and methodological issues in qualitative research in older adults with major neurocognitive disorder (Hellstrom et al., 2007; Beuscher and Grando, 2009) were applied. The interviewer had a respectful attitude, made eye contact when appropriate, used a calm voice, and avoided contradicting participants’ statements or asking about details. The interviewer considered the communication challenges – such as word-finding difficulties, abstract reasoning, memory deficits, fluctuating awareness, attention, and concentration – by allowing sufficient response time, and gently redirecting the dialogue when needed.

### Montreal Cognitive Assessment

Participants completed the Montreal Cognitive Assessment (MoCA) before the exergame try-out. The MoCA is a paper and pencil test that assesses memory, language, executive functions, visuospatial skills, attention, concentration, abstraction, calculation, and orientation. The scores range from 0 to 30, with higher scores indicating better cognitive functioning. The MoCA has good construct validity (*r*-values range from 0.46 to 0.75) (Freitas et al., 2012), inter-rater reliability (*r* = 0.97), test–retest reliability (*r* = 0.88) and internal consistency (Cronbach’s α = 0.89) in older adults with MNCD (De Roeck et al., 2019).

### Short Physical Performance Battery

Prior to the exergame try-out, the SPPB was administered. The SPPB assesses gait speed, balance, and lower limb strength (Guralnik et al., 1994; Fox et al., 2014). It is composed of three subtests; a standing balance test, a short 4-m walk at usual pace (Kim et al., 2016), and 5 chair rises. The maximal total score is 12 and higher total scores indicate a better lower extremity functioning. The reliability of the SPPB is high in older adults with MNCD, with intraclass correlation coefficient values ranging between 0.82 and 0.92 (Guralnik et al., 2000; Ostir et al., 2002; Olsen and Bergland, 2017). The SPPB is highly predictive for disability in older adults (Guralnik et al., 2000) and the internal consistency is acceptable (Cronbach’s α = 0.76) (Guralnik et al., 1994).

### Data Analysis

Continuous data were tested for normality using the Shapiro–Wilks test and found to be normally distributed. Descriptive statistics (means and standard deviations) were used to provide general information on the study outcomes. To aid management and analysis of the think aloud method, field notes and interviews, the NVivo 12 Microsoft software for qualitative data analysis (QSR International Pty Ltd., VIC, Australia) was used (McLafferty and Farley, 2006; Zamawe, 2015). Individual interviews, field notes and think-aloud data were transcribed in Microsoft Word format and afterward inserted into one project in NVivo 12. A thematic analysis of this project was performed through six consecutive steps (Braun and Clarke, 2006, 2014). The first step in the analysis consisted of repeatedly reading the transcripts and listening to the interview recordings to obtain further information from the tone of voices and pauses. Next, initial codes were created by open coding – the process of indexing or categorizing the text to establish a framework of related ideas. Subsequently, the residual data were examined through axial coding, which is relating codes to possible sub-codes to form a more precise and complete explanation. Codes with similar content were merged. The categories that remained were further interpreted and abstracted into the remaining themes. Although the observations and interview transcripts in NVivo 12 formed the primary data set, the SUS scores were investigated separately. After these steps, a composite description of the participants’ perspectives on using the exergames was written, while using quotes to underpin the interpretation. A backward stepwise multivariable regression analysis was performed to evaluate independent variables (i.e., age, gender, MoCA and SPPB total score) explaining the variance in SUS. To test for multicollinearity, a variance inflation factor was computed for each independent variable in the model. Values above 3 were used to indicate a multicollinearity problem in the model. A priori, a two-sided level of significance was set at *P* < 0.05. Statistical analysis was performed using the statistical package SPSS version 28.0 (SPSS Inc., Chicago, IL, United States).

## Results

### Participants

Thirty-three of the 147 residents in the long-term care facility were eligible. Main reasons for exclusion were limited comprehension due to an advanced stage of MNCD, the use of a wheelchair, or being bedridden. Eleven residents refused as they were not interested. Therefore, in total 22 participants were enrolled in the study. They had a mean age of 84.3 ± 5.5 (70–95) years, a SPPB score of 7.5 ± 3.2 (1–12), and a MoCA score of 11.9 ± 4.4 (2–19). 81.8% of the participants were female. Table 1 gives an overview of the characteristics of the included participants. A more detailed description of the participants’ individual characteristics is provided in Table 2. None of the participants suffered adverse events during or after the exergame session.

**TABLE 1:** Characteristics of the included participants (*n* = 22).

Age, median	85 (70–95)
Women, n (%)	18 (81.8%)
Montreal Cognitive Assessment (0–30), mean ± standard deviation	11.9 ± 4.4 (2–19)
**Diagnosis**	
- Alzheimer’s disease, *n* (%)	10 (45.5)
- Vascular dementia, *n* (%)	5 (22.7)
- Neurocognitive disorder not otherwise specified, *n* (%)	6 (27.3)
- Lewy body disease, *n* (%)	1 (4.5)
**Comorbidities**	
- Diabetes, *n* (%)	9 (40.9)
- Heart failure, *n* (%)	5 (22.7)
- Dizziness, *n* (%)	9 (40.9)
- Urinary incontinence, *n* (%)	9 (40.9)
- Mild back pain, *n* (%)	4 (18.2)
**Indoor mobility**	
- 4-wheeled walker, *n* (%)	4 (18.2)
- Single-point walking cane, *n* (%)	3 (13.6)
- No walking aid, *n* (%)	15 (68.2)
**Fear of falling**	
- Never, *n* (%)	12 (54.5)
- Sometimes, *n* (%)	3 (13.6)
- Regularly, *n* (%)	5 (22.7)
- Always, *n* (%)	2 (9.1)
**Physical activity level before participation**	
- No physical activities, *n* (%)	8 (36.3)
- One walking session per week, *n* (%)	7 (31.8)
- One to three walking sessions per week, *n* (%)	5 (22.7)
- More than three walking sessions per week, *n* (%)	2 (9.1)
- One gymnastics session per week, *n* (%)	1 (4.5)

**TABLE 2:** Individual characteristics of the included participants.

Subject ID	Age	Gender	MoCA	Diagnosis	Mobility	SPPB
1	88	F	19	AD	No aid	1
2	87	F	4	AD	No aid	8
3	86	F	13	AD	No aid	7
4	87	F	2	AD	No aid	2
5	70	F	15	AD	No aid	12
6	80	F	7	NCD NOS	No aid	11
7	88	F	8	AD	Walker	2
8	83	F	14	VD	Cane	7
9	82	F	15	NCD NOS	Cane	7
10	80	M	11	LBD	No aid	8
11	85	F	8	AD	No aid	7
12	82	F	15	VD	No aid	11
13	95	M	17	NCD NOS	Walker	5
14	77	F	17	NCD NOS	No aid	9
15	89	F	7	VD	No aid	9
16	90	F	13	AD	Walker	4
17	84	F	12	AD	No aid	6
18	78	M	16	NCD NOS	No aid	11
19	85	F	14	VD	Walker	11
20	81	F	13	AD	No aid	11
21	92	M	12	VD	Cane	9
22	85	F	9	NCD NOS	No aid	6

*AD, Alzheimer’s disease; F, female; LBD, Lewy body disease; M, male; MoCA, Montreal Cognitive Assessment (total scores range from 0 to 30 with lower scores indicating more cognitive impairment); NCD NOS, Neurocognitive disorder not otherwise specified; SPPB, Short Physical Performance Battery (total scores range from 0 to 12 with lower scores indicating a higher risk and a score lower than 10 indicates one or more mobility limitations); VD, vascular dementia.*

### System Usability Scale

The mean rating given to the VITAAL exergame by participants was 57.8 (*SD* = 12.3) with total scores ranging from 37.5 to 90.0. The mean SUS score of 57.8 corresponds to a system that is considered ok to good (Sauro, 2011). The SUS scores per participant are provided in Table 3.

**TABLE 3:** System usability scale scores.

Participant ID	Q1	Q2	Q3	Q4	Q5	Q6	Q7	Q8	Q9	Q10	Sum	Score
1	4	3	4	4	4	2	4	2	2	4	23	57.5
2	4	4	3	5	5	3	4	2	4	1	25	62.5
3	1	2	5	5	3	3	4	3	4	3	21	52.5
4	4	4	2	5	4	4	4	3	2	5	15	37.5
5	1	1	4	5	3	2	5	1	5	2	27	67.5
6	4	2	4	5	4	4	4	2	4	3	24	60
7	1	2	4	5	3	3	2	2	2	4	16	40
8	4	2	4	5	4	4	5	2	5	3	26	65
9	5	2	4	5	4	2	2	4	4	5	21	52.5
10	4	4	4	1	4	2	4	4	2	1	26	65
11	4	2	4	4	5	4	2	2	5	2	26	65
12	4	1	4	2	4	3	3	1	4	2	30	75
13	4	3	4	4	4	3	4	2	4	2	26	45
14	5	2	5	2	5	2	4	1	5	1	36	90
15	2	2	4	2	3	3	4	2	3	2	25	62.5
16	4	4	3	5	3	2	2	2	5	2	22	55
17	3	4	4	4	4	3	4	4	5	1	24	60
18	2	2	2	5	4	3	2	2	4	2	20	50
19	4	2	3	4	4	4	5	2	2	1	25	62.5
20	2	3	2	5	4	2	2	3	2	2	17	42.5
21	4	4	4	2	4	2	4	4	5	4	25	62.5
22	1	2	2	5	4	2	3	2	2	4	17	42.5

*1: strongly disagree, 2: disagree, 3: neutral, 4: agree, 5: strongly agree, Q: SUS question*

*Questions:*

*(1) I think that I would like to use this system frequently.*

*(2) I found the system unnecessarily complex.*

*(3) I thought the system was easy to use.*

*(4) I think that I would need the support of a technical person to be able to use this system.*

*(5) I found the various functions in this system were well integrated.*

*(6) I thought there was too much inconsistency in this system.*

*(7) I would imagine that most people would learn to use this system very quickly.*

*(8) I found the system very cumbersome to use.*

*(9) I felt very confident using the system.*

*(10) I needed to learn a lot of things before I could get going with this system.*

### Thematic Analysis

The collective analysis of the interviews, the think aloud method and the field notes revealed five main themes which describe the experiences of the participants: (1) perceived user friendliness and acceptability of the exergames; (2) interactional experience; (3) motivational factors; (4) training; and (5) risks.

#### Perceived User Friendliness and Acceptability of the Exergames

##### Attitude Toward Using the Exergame Device

All participants liked the minigames and experienced enjoyment while playing them (*n* = 22, 100%). Ten participants stated that they would be interested in using the minigames in the future, next to traditional activities in the long-term care facility (45.5%).

*I would like that because I feel that it’s good for my lower vertebrae (P9)*.

Five participants were not sure about using the exergame device in the future (22.7%).

*I would have to think about that… it’s something peculiar, isn’t it? (P17)*

Six participants would not be interested in exergaming in the future (27.3%).

*I prefer to go walking instead of exergaming (P3)*.

##### Ease of use and Understandability of the Instructions

Participants were all assisted by the facilitator with the setup of the system and the positioning of the sensors. They were not expected to do this independently. Participants experienced difficulties in navigating between different minigames. The minigames were depicted at the home screen and could be accessed by performing steps in the right direction. A representation of the home screen can be found in Supplementary Material 2. Participants needed verbal guidance to perform steps in different directions to access the various minigames from the home screen. They found it difficult to understand that they were supposed to perform stepping movements to access the minigames. In addition, they did not understand the minigame instructions and needed supplementary explanation from the facilitator. Verbal guidance was needed in all participants during game performance for assistance in navigating between the minigames and game explanation (*n* = 22, 100%). Three participants kept looking at their feet and had to be reminded to look up to the screen to see the game interface (13.6%). Four participants initially moved their hands to the screen (instead of using whole-body movements to interact with the game) because they did not understand that they needed to perform steps to play the minigame (18.2%). Nine participants were not able to play the minigame without constant verbal guidance (40.9%).

##### Sensor Application

Eight participants said that the sensors were user-friendly (36.4%). Some participants said that they forgot that they were even wearing sensors (*n* = 3, 13.6%).

*The sensors didn’t bother me, I wasn’t even aware that they were attached to my feet (P5)*

Two participants stated that they expected that they would not be able to apply the sensors to their feet themselves (9.1%).

*I wouldn’t be able to apply the sensors myself (P4)*.

##### Sensor Reactivity

One participant accidentally exited the minigames ten times, because the sensors often falsely perceived her steps as calf raises, which is also the movement required to go to the menu (4.5%). In five participants, the exergame did not respond to a good execution of the calf raises because of processing delays (22.7%). Two participants accidentally exited the game because their movements were perceived as movements required to exit the game (9.1%).

One participant performed her steps very slowly; when she placed her foot back in the center, this was perceived as an opposite direction sidestep by the device (4.5%). Often, the steps were not detected at all, or with a delay. In most participants, the sensors did not correctly process steps, so the facilitator had to assist by clicking the arrows on the keyboard to play the minigames and navigate through the minigames.

##### Technical Problems

Apart from the problems with the sensors, participants did not experience any technical problems with the exergame solution while playing.

##### Physical Limitations

Five participants were not able to perform the calf raises on both feet to exit the game or to go back to the menu without assistance of the facilitator (22.7%). Seven participants needed extra support from the facilitator, a walker, or a walking cane to play the minigames (13.6%).

The step backward was regarded as the most difficult step direction, because this action requires a good equilibrium.

It was also hard for participants with hearing difficulties to understand the game instructions given by the facilitator.

##### Mental Effort

For most participants, the minigames were cognitively more challenging than physically. Some had difficulties staying focused on the exergames.

Twelve participants said that exergaming was mentally exhausting (54.5%) and nine said that it was not (40.9%).

*It was a bit mentally challenging because it was all new to me (P20)*

*It was necessary to keep your attention (P6)*.

#### Interactional Experience

##### Feedback

Participants enjoyed receiving feedback from the game (*n* = 17, 77.3%) and some even laughed out loud when positive feedback was given (*n* = 6, 27.3%).

*Oh, it feeds your ego of course (P12)*

*It is encouraging (P19)*

*I realized that I was good at it due to the score (P14)*

Sometimes participants were performing well but received negative feedback because their intended steps were not properly evaluated by the system (*n* = 22, 100%).

##### Multidirectional Steps

Some participants were not able to link the steps to the directions in the minigames (*n* = 5, 22.7%). For example, it was hard to link the backward step with downstairs in the library game. For several participants, it was difficult to navigate between the minigames, so the facilitator had to assist by pressing the arrows on the keyboard. Some participants had a hard time learning to just tap their feet and took a whole sidestep with both feet instead, causing the exergame device to react falsely (*n* = 9, 40.9%).

##### Avatar Interaction

Participants were able to associate themselves with their avatar. They enjoyed seeing their avatar and found it easy to imitate the avatar’s movements. One participant even scratched her hair when her avatar did (4.5%). However, the home screen avatar “Vita” was recognized as a dog by one participant (4.5%). A picture of the avatar Vita can be found in Supplementary Material 3. The squatting avatar in the outdoor game especially was perceived as enjoyable and helpful. A picture of this avatar, which displays a full human body, can be found in Figure 1.

#### Motivational Factors

##### Exergame Motivation

Most of the participants found exergaming to be motivating (n = 19, 86.4%).

*It motivated me because the exercises were easy to perform (P9)*

*It is very healthy, and you must know that I am very patient, but lately (due to COVID-19 restrictions) we are not allowed to do gymnastics anymore (P11)*

*I don’t exercise enough, and you should exercise, exercise, exercise (P14)*

However, four participants said that they were already active enough and did not need exergames to motivate them to be physically active (18.2%).

*The exergame did not motivate me because everything hurts. I never really enjoy physical activity. I have done enough in my life already (P7)*.

##### Enjoyment and Positive Emotions

All participants experienced having fun and smiled while exergaming (100%). Some stated that they just liked being invited to go outside their living unit and enjoyed the distraction. Participants were enthusiastic about the exergames. Exergaming evoked memories and three participants spontaneously started talking about past experiences with physical activity (13.6%). Eight participants felt that they excelled in it (36.4%).

##### Engagement

Participants spontaneously started talking about healthy food while playing the healthy food game (*n* = 4, 18.2%). Seventeen participants experienced feeling “in” the game regularly (77.3%).

*In the beginning, I had to listen carefully to understand the instructions, but afterwards I experienced it (P11)*.

##### Long-term Acceptability

Fourteen participants said that they expected that the exergames would still be nice, even after they would have played them several times (63.3%). Two of them argued that this would be the case, provided that the games would become more difficult over time.

*I think it would be even more nice, because then you really know how it works and it’s easier (P16)*

*I will become better at it and these are movements that you usually don’t do; you never step backwards and it’s actually very beneficial for your balance (P8)*.

##### Game Design: Sounds and Images

Participants liked the appearance of the minigames. They enjoyed the music that was played during the exergames and while navigating between the minigames. Two participants (9.1%) spontaneously started dancing to the exergame music.

#### Training Modalities

##### Exergame Intensity

Most of the participants stated that the minigames were of low intensity levels (*n* = 13, 59.1%). However, three participants said that performing the squats was particularly difficult and needed to rest in between (13.6%). Two participants said that the walk to the exergame room was already exhausting for them (9.1%).

*I would prefer to do more high intense exercises (P14)*

*I was already fatigued and to perform this on top of that… It’s particularly exhausting for my eyes (P7)*.

##### Training Duration

All participants said that the duration of the exergame session was good (*n* = 22, 100%).

##### Feeling Safe

Although all participants felt safe during exergaming (*n* = 22, 100%), four stated that they were extra careful not to fall (18.2%).

*I am not afraid of falling, but I try to be careful not to fall (P22)*.

#### Risks

##### Fall Risk

The facilitator always individually guided the participant and there were no fall incidents. Two participants indicated that the floor on which they were standing was slippery (9.1%). This feeling was augmented because the sensors were attached to the feet with fabric straps that slid on the floor very easily.

##### Negative Emotions

Four participants felt confused because they didn’t understand the instructions of the games (18.2%). One participant said that the games at first seemed to be childish.

*It might seem childish at first, but it’s not (P10)*.

### Variables Explaining the Variance in the System Usability Scale Scores

None of the variables (i.e., age, gender, MoCA and SPPB total score) included in the backward stepwise multivariable regression analysis had a variance inflation factor of more than 3 and needed to be removed. Only the variance in the SPPB total score remained a significant predictor of the variance in the SUS score and explained 21.9% of the variance (unstandardized B coefficient = 1.80, standard error = 0.76, standardized β coefficient = 0.47, *t* = 2.37, *P* = 0.028; constant: unstandardized B coefficient = 44.5, standard error = 6.12, *t* = 7.26, *P* < 0.001).

## Discussion

The primary aim of this study was to evaluate the usability of the VITAAL stepping exergame prototype in residential older adults with MNCD. Overall, the mean SUS score given to the VITAAL exergame was 57.8, which corresponds to a system usability that is ok to good. The SUS scores also correspond to the observations of the facilitator and the content of the interviews. Five main themes emerged from the thematic content analysis: (1) perceived user friendliness and acceptability of the exergames; (2) interactional experience; (3) motivational factors; (4) training modalities; and (5) risks. There were no adverse events nor dropouts. We will discuss all the themes more in detail.

A first theme was the perceived user friendliness and acceptability. Overall, the VITAAL exergame prototype was well accepted by participants. Participants were always assisted by the facilitator with the setup of the exergame and the correct application of the sensors to the feet. However, some difficulties regarding user friendliness and acceptability were reported. For example, all participants experienced difficulties in understanding at least some of the game instructions. Additional verbal guidance from the facilitator was therefore needed in all participants. Nine participants (40.9%) were not able to play the game without constant verbal guidance of the facilitator. Some participants also needed extra physical support, their walker, or their walking cane during exergame performance (13.6%). From all these findings, the importance of prompts given by the facilitator when learning people with MNCD to use new technologies becomes evident. Previous research already demonstrated that verbal prompts (i.e., words used to provide instruction), gesture prompts (i.e., steps modeled using physical actions), and physical assistance (i.e., any physical intervention) are essential for people with MNCD when learning to use motion-based technologies such as exergames (Dove and Astell, 2019). Recently, a call was made to formulate guidelines for researchers, clinicians, care providers, and families on how to start implementing these new technologies in the rehabilitation of people with MNCD (Dove and Astell, 2017b). One should, however, be aware that difficulties regarding understanding the game instructions and game navigation in the current study might also be due to the fact that participants only had one try-out session. One session might not be sufficient to get familiarized with the instructions and execution of the exergames. Another aspect related to perceived user friendliness and acceptability was that the exergames were cognitively challenging for most participants. Twelve participants reported that exergaming was mentally exhausting (54.5%). It would be interesting to examine in the updated prototype how to adapt the game to the performance level and needs of the individual. This adaptation will likely also increase the usability. Our backward stepwise multivariable regression analysis demonstrated that in particular the variance in lower extremity functioning explained the variance in usability, with lower extremity functioning being associated with a lower perceived usability. It has been previously reported that in people with MNCD, the acceptance and user friendliness of an exergame device strongly depends on the task itself and the perceived competence (Vallejo et al., 2016). The current usability study therefore confirms previous findings that in order to create an exergame-based rehabilitation program, it is essential to consider the usability of the involved device, the persons’ abilities and the motivations to play of the target population (Vallejo et al., 2016). For example, one in five participants were also not able to perform calf raises on both feet to exit the minigame or to go back to the menu without assistance of the facilitator. The final design should therefore consider these physical limitations, in particular for old-aged populations at risk for falling. In addition, participants were not able to navigate between the minigames because the sensors were not responding correctly to the calf raises (100%). When the sensors were not working well and the system falsely provided negative feedback, the facilitator tried to solve this by giving appropriate positive verbal feedback. A possible explanation might be that Bluetooth and Wi-Fi devices, such as a smartphone or a tablet from nearby staff in the long-term care facility, might have caused interference in the connection between the sensors and the software. Therefore, it is recommended to examine the difficulties with sensor reactivity and to solve them before using the prototype in a future trial. Despite these issues, the sensors were perceived as user-friendly (36.4%), and participants did not report experiencing any technical problems with the exergame while playing (100%). Nearly half of the participants also expressed a wish to continue exergaming in the future, supplementary to their traditional activities in the long-term care facility (45.5%).

A second theme was the interactional experience with the minigames, which was overall positive as well. Participants particularly liked the squatting avatar in the Tai Chi-inspired strength training game. Participants found it helpful to imitate the avatars’ movements because they were able to associate themselves with their avatar. A reason for this might be that in this game, the avatar was displayed as a full human body. This contrasts with the avatar of the home screen and avatar in the other minigames, which was more abstract and did not resemble a human being. For application in our population, it might be recommended to adapt avatars to resemble a more human-like avatar. Concerning the audio-visual feedback, the VITAAL prototype exergame focused on positive feedback and this was greatly appreciated by the participants. They enjoyed receiving feedback (77.3%) and laughed out loud when positive feedback was given (27.3%). Positive feedback is commonly recommended in order to promote motor skill learning and neurorehabilitation of motor functions (Vassiliadis et al., 2021). Despite the positive interactional experience, some interactional issues were detected as well. For example, participants had difficulties learning to just tap their feet and took a whole sidestep with both feet instead, causing the exergame to react falsely (40.9%). Moreover, some participants initially pointed their fingers to the screen or tried to grab items displayed on the screen, instead of performing steps to control the minigames (18.2%). This was easily solved by extra verbal guidance of the facilitator. Related to the interaction experience, although the VITAAL exergame is conducive for single-player activities, future research should explore differences in interactional experience of the VITAAL exergame between individual and group settings. Previous studies in people with MNCD already demonstrated that using single-player exergame technology in a group setting fosters an encouraging and supportive environment which further contributes to the leisure experience. Using motion-based technology in a group setting creates opportunities for social interaction amongst group members and between the players and facilitators (Fenney and Lee, 2010; Dove and Astell, 2017a). The value of well-trained facilitators and the verbal and non-verbal communication between the facilitator and institutionalized players with MNCD has been discussed previously. Researchers underlined the importance of enjoyment, empathetic communication in both ways, the use of praise, and the development of social roles (Yamaguchi et al., 2011).

Third, with regards to the motivational factors, most of the participants found exergaming to be motivating (86.4%). All participants experienced enjoyment and fun while playing the exergames (100%). Our data confirm previous trials showing that exergames can be engaging and motivating in people with moderate cognitive impairments (Ben-Sadoun et al., 2015, 2016). This finding is important since drop-out is a major problem in physical activity programs for people with MNCD (Forbes et al., 2015) and experiencing enjoyment is a strong predictor for training adherence in exergame programs for old-age populations (Wiemeyer and Kliem, 2012). Most participants experienced feeling “in” the game regularly (77.3%). This feeling possibly reflects the experience of participants being in their “flow zone,” a feeling of complete and energized focus in order to improve the enjoyment and learning experience (Robert et al., 2014). Participants in our study stated that the game design was engaging. They also enjoyed the music while playing the exergames and when they were navigating between the minigames. Two participants spontaneously started dancing to the exergame music (9.1%). The healthy food game that focused on inhibition control was preferred by all participants and some of them spontaneously started talking about healthy food while playing (18.2%). We believe that this reflects a proper choice of minigame themes and a possible relation of their game experience to daily life situations.

A fourth theme handled the training modalities. Most of the interviewees stated that they experienced the intensity of the exergames as low (59.1%). However, executing the squats in the Tai Chi-inspired strength training game was perceived as more intense, and some participants sat down on a chair during the breaks (13.6%). Since older adults with MNCD are less able to describe their perceived exertion validly due to impaired judgment, awareness, and insights as well as increased communication difficulties, it might be hypothesized that an automatic adaptation of the exergames to the individual needs and performance of the player will increase the usability of the VITAAL exergame even further. The duration of the exergame session was 30 min and this was unanimously perceived as good (100%). It should be considered, however, that once the exergame automatically adapts to the individual’s needs and performance, the intensity level might increase. Consequently, 30 min might be too long for our sedentary population. In such a case, gradual progression of exergame play time should be warranted and the 30 min should be a target that should only be reached following a skilling-up phase (Rogan et al., 2012).

A fifth and final theme included the risks regarding the use of the VITAAL exergame. Although all participants felt safe during exergaming (100%), some explained that they were extra attentive not to fall (18.2%). The facilitator considered five participants as having a higher risk of falling, although this was not objectively represented (Shimada et al., 2011). The sensors were attached to the feet with fabric straps that slid on the floor very easily. Depending on the floor, this might increase the risk of falling during exergame performance. Therefore, in future exergame trials it is recommended that the facilitator is aware of the potential risk of falling and takes the necessary precautions. The VITAAL exergame occasionally evoked negative emotions such as confusion when the participants did not understand the instructions of the minigames (18.2%). Therefore, the facilitator assisted by explaining the game instructions in a friendly way. This also underscores the advantage of the one-on-one guidance during exergaming.

Some limitations of this study should be considered. First, the current study was limited to only one long-term care facility in Belgium, so the findings may have limited generalizability to other settings and countries. Second, only residents who were willing to participate, in other words, those who were more interested in technology and physical activity than the average person with MNCD, were included. This limits the generalizability to all older adults with MNCD. It has been previously stated that only a minority of long-term care residents with a MNCD are suitable for inclusion in an exergame training program. A factor that might influence acceptance of exergames is the level of cognitive functioning. More specifically, residents with more severe cognitive impairments were more likely to reject exergame training (Ulbrecht et al., 2012). A third limitation of the study was that more female (81.8%) than male (18.2%) participants were included. A reason for this might be that women are at greater risk for developing Alzheimer’s disease (Podcasy and Epperson, 2016) and more women than men are living in long-term care facilities in Belgium (Vlaamse overheid Statistiek Vlaanderen, 2018). Fourth, the SUS has not been validated in older adults with MNCD yet (Gibson et al., 2016). Therefore, these results were interpreted in conjunction with the data from the observations and interviews. Fifth, although the think aloud method has previously been applied in research in older adults with MNCD (Ratcliffe et al., 2019), for most participants, talking out loud while exergaming was complex. This is in line with a preliminary usability study in older adults with MNCD stating that participants experienced difficulties in verbalizing and narrating their experiences, even when prompted and reminded to do so during completion of the tasks (Gibson et al., 2016). Sixth, the results may have been influenced by social desirability bias. This was considered during the interviews by for example actively asking about negative impressions. Finally, the views and opinions of the caregivers were not assessed. It would be of added value to actively involve caregivers and ask about their opinions regarding the exergame device and technological features as well.

Despite these limitations, some strengths should be acknowledged. The number of participants allowed for a rich data collection of experiences and usability opinions. Although the exergame prototype did not adapt to the individual needs of the participant, which might be considered as a limitation from a clinical perspective, it allowed us to investigate a standardized exergame training session. Moreover, interviews were performed directly following the exergame try-out and in the same room, which stimulated participants’ recall of the events and experiences during the exergame session. Also, the interviewer was the same person who facilitated the participants’ exergame session, and so they were familiar with each other. Furthermore, the facilitator always attempted to adopt a neutral body language, in order not to influence the participants’ responses.

## Conclusion

Based on the current findings, it can be concluded that the VITAAL exergame prototype is considered useful and entertaining by residential older adults with MNCD. Technical issues concerning the sensor reactivity and the challenges regarding the minigame navigation and instructions should be addressed before the prototype can be implemented in a longitudinal trial. Subsequently, investigating whether this exercise solution can overcome sedentary behavior in this population seems warranted.

## Data Availability Statement

The original contributions presented in the study are included in the article. Further inquiries can be directed to the corresponding author.

## Ethics Statement

The studies involving human participants were reviewed and approved by the Medical Ethics Committee of UZ Leuven (registration: S63304/B322201941828). No compensation for participation was given. The participants provided their written informed consent to take part in this study.

## Author Contributions

NS developed the research question under the lead of DV, MV, and EB. NS established the concept and design while DV, MV, and EB acted as methodological council. NS conducted data processing, analysis, and interpretation of the results with edits and improvement by all authors and produced a first version of the manuscript. All authors have revised and approved the final manuscript.

## Conflict of Interest

The authors declare that the research was conducted in the absence of any commercial or financial relationships that could be construed as a potential conflict of interest.

## Publisher’s Note

All claims expressed in this article are solely those of the authors and do not necessarily represent those of their affiliated organizations, or those of the publisher, the editors and the reviewers. Any product that may be evaluated in this article, or claim that may be made by its manufacturer, is not guaranteed or endorsed by the publisher.
