# Supplementary material for: The VITAAL Stepping Exergame Prototype for Older Adults With Major Neurocognitive Disorder: A Usability Study
Source: Front Aging Neurosci. 2021 Nov 4;13:701319. doi: 10.3389/fnagi.2021.701319 (PMC8600328; doi:10.3389/fnagi.2021.701319)
Supplement: Supplementary file 1 [file Data_Sheet_1.docx]

Supplementary Material 1: interview guide

**General**1. What do you remember the most regarding the exergame?

2. How did you feel while playing the exergames?

**Game**1. Which game did you like most? Why?

2. Was it difficult for you? What game or situation?

3. What did you think of the view or sounds of the games?

**VITAAL Exergame**1. Where the sensors easy to use?

2. Could you control the games easily?

3. Were there any technical problems with the VITAAL Exergame? If so, what were the problems?

**Body & mind**1. Did the movement feel good to you?

2. Did you feel like you were "in" the game?

3. Have you always realised how good/bad you were in the game?

**Motivation**1. Has the game motivated you to move? How and why/why not?

2. Do you think the game would still be fun after you played it a few times?

3. Could you imagine using such games in addition to the traditional range of movement in the Wingerd?

4. How did you experience the feedback during the game?

**Training**1. What went well while playing?

2. Did you find it physically intensive?

3. Was it mentally exhausting?

4. How did you experience the duration of playing?

5. Did you always feel safe during training? Were you afraid of falling? When exactly?

**Other**

1. Is there anything else you would like to say?

2. Do you have ideas/wishes for the future development of the games?
