# Supplementary material for: The VITAAL Stepping Exergame Prototype for Older Adults With Major Neurocognitive Disorder: A Usability Study
Source: Front Aging Neurosci. 2021 Nov 4;13:701319. doi: 10.3389/fnagi.2021.701319 (PMC8600328; doi:10.3389/fnagi.2021.701319)
Supplement: Supplementary file 2 [file Data_Sheet_2.docx]

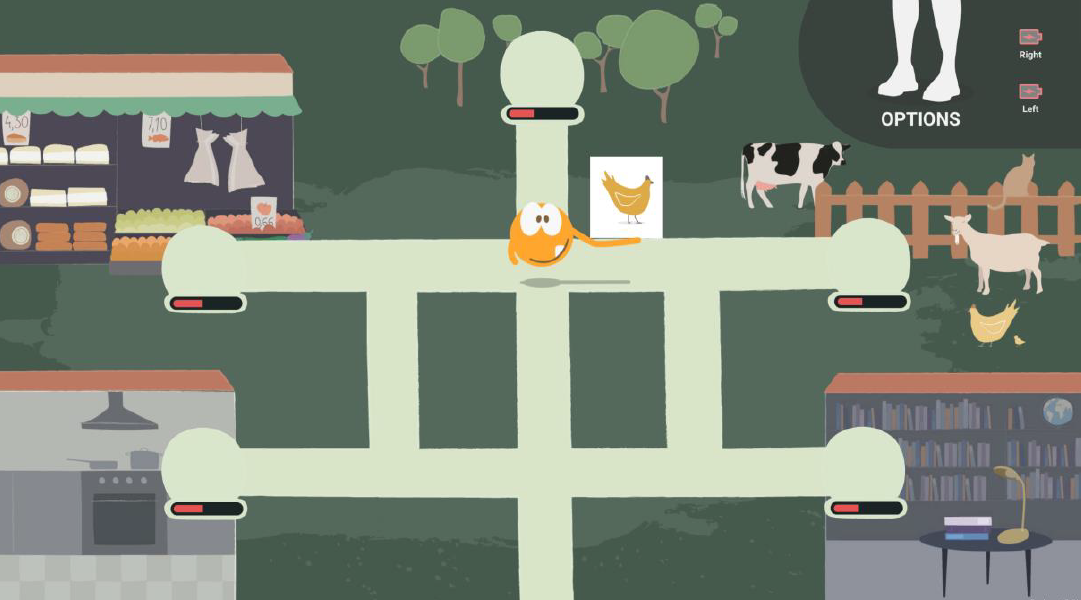


Supplementary Material 2: VITAAL home screen: needs and priorities of actions are indicated in the bars
