# Supplementary figures and images for: The VITAAL Stepping Exergame Prototype for Older Adults With Major Neurocognitive Disorder: A Usability Study
Source: Front Aging Neurosci. 2021 Nov 4;13:701319. doi: 10.3389/fnagi.2021.701319 (PMC8600328; doi:10.3389/fnagi.2021.701319)

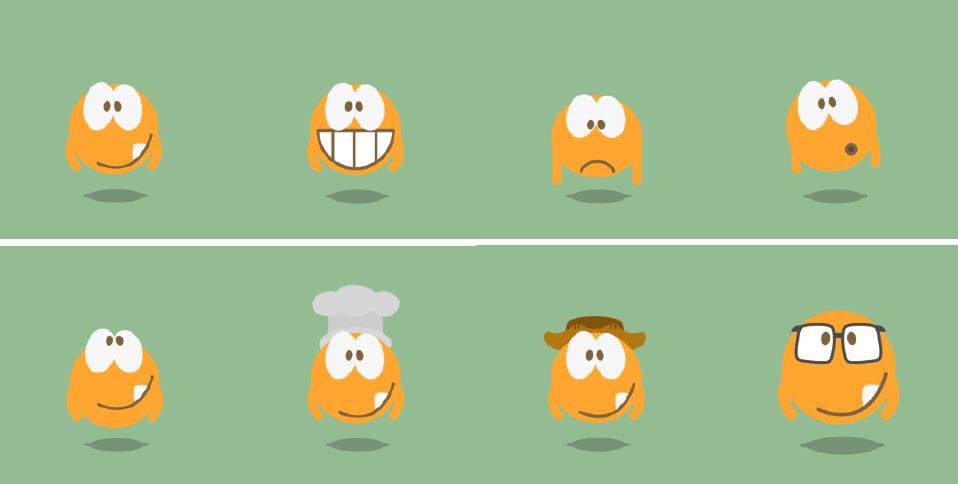


Supplementary Material 3: The different looks of avatar Vita

Supplement: Supplementary file 3 [file Data_Sheet_3.docx]
